# Reliability of crowdsourced data and patient-reported outcome measures in cough-based COVID-19 screening

**DOI:** 10.1038/s41598-022-26492-5

**Published:** 2022-12-20

**Authors:** Hao Xiong, Shlomo Berkovsky, Mohamed Ali Kâafar, Adam Jaffe, Enrico Coiera, Roneel V. Sharan

**Affiliations:** 1 Centre for Health Informatics, Australian Institute of Health Innovation, Macquarie University, Sydney, Australia; 2 Department of Computing, Macquarie University, Sydney, Australia; 3 School of Women’s and Children’s Health, Faculty of Medicine, University of New South Wales, Sydney, Australia

**Keywords:** Respiratory tract diseases, Respiratory signs and symptoms

## Abstract

Mass community testing is a critical means for monitoring the spread of the COVID-19 pandemic. Polymerase chain reaction (PCR) is the gold standard for detecting the causative coronavirus 2 (SARS-CoV-2) but the test is invasive, test centers may not be readily available, and the wait for laboratory results can take several days. Various machine learning based alternatives to PCR screening for SARS-CoV-2 have been proposed, including cough sound analysis. Cough classification models appear to be a robust means to predict infective status, but collecting reliable PCR confirmed data for their development is challenging and recent work using unverified crowdsourced data is seen as a viable alternative. In this study, we report experiments that assess cough classification models trained (i) using data from PCR-confirmed COVID subjects and (ii) using data of individuals self-reporting their infective status. We compare performance using PCR-confirmed data. Models trained on PCR-confirmed data perform better than those trained on patient-reported data. Models using PCR-confirmed data also exploit more stable predictive features and converge faster. Crowd-sourced cough data is less reliable than PCR-confirmed data for developing predictive models for COVID-19, and raises concerns about the utility of patient reported outcome data in developing other clinical predictive models when better gold-standard data are available.

## Introduction

COVID-19 disease (hereafter, COVID) is a respiratory disease caused by the severe acute respiratory syndrome coronavirus 2 (SARS-CoV-2)^1^. COVID was declared a pandemic in March 2020^2^ and, as of September 2022, there have been more than 600 million confirmed cases of COVID worldwide, with more than 6.5 million deaths attributed to the disease^3^. Mass testing, combined with isolation and contact tracing, are pivotal for mitigating the spread of the pandemic^4–8^. Reverse transcription polymerase chain reaction (PCR) is the common gold standard for testing SARS-CoV-2^9^. SARS-CoV-2 tests can be grouped into molecular, antigen, and antibody tests^10,11^. Molecular tests detect the genetic material of the virus and antigen tests detect the virus proteins. Antibody tests, such as serology, look for antibodies created by the immune system in response to the virus. PCR tests are invasive, and require body samples such as throat swabs or blood samples. However, supply chain issues and limited capacity of testing facilities may extend the waiting time and render testing cumbersome^12,13^.

Cough is a common symptom of various respiratory infections, including COVID. Respiratory tract infections can produce unique cough and breathing sounds, such as barking cough in croup^14^, hacking cough and whoops in pertussis^15^, or crackles in pneumonia^16^. While the cough and breathing sounds of COVID are not well studied yet, an early report observed dry cough in about two-thirds of confirmed cases^17^. Although not distinguishable by clinicians, it has been hypothesised that recent developments in sound processing and machine learning warrant the development of computational methods for detection of COVID cough^18,19^.

Several cough-based predictive algorithms for rapid screening of COVID have been recently developed using crowdsourced data for training^10,20^. Due to data access constraints, these models are mostly developed using patient-reported data which are seen as a promising alternative to PCR confirmed data. For example, Brown et al. deployed a combination of handcrafted and transfer learning-based features for detecting subjects with COVID^21^. The cough recordings were crowdsourced using smartphone apps and the Web. Laguarta et al. also crowdsourced the data collection and employed deep learning methods for classification^22^. Crowdsourced datasets are also exploited in other studies^23–26^.

While promising results have been achieved, we question the validity of results achieved using such crowdsourced patient-reported training data, stemming from the unreliable nature of the data. Patient-reported status is substantially easier to obtain than the PCR-confirmed one; however, it is prone to noise and confounding associated with, e.g., inconsistency in recording device positioning, differences in the sound processing technologies, background noises, disease symptoms and co-morbidities, and disease progression variabilities. Also, patient-reported COVID status used as the training labels can be unreliable due to subjects misinterpreting symptoms or incorrectly estimating their infective status.

To the best of our knowledge, Bagad et al. is the only work where COVID status was confirmed using PCR at testing facilities and isolation wards rather than was patient-reported^27^. They used various datasets for training their cough-based COVID detection model, achieving a substantially lower accuracy than studies using patient-reported COVID status as the data labels^21,22^. This suggests a disparity in the performance of machine learning based COVID classification models using cough data of subjects using PCR confirmed and self-reported infective status.

With growing interest in harnessing crowdsourced health data, this present study sets out to investigate the reliability of using crowdsourced cough data for developing COVID screening algorithms. To this end, we experimentally compare the performance of several existing machine learning and deep learning models trained using cough sounds, where the subjects’ infective COVID status is either PCR-confirmed or patient-reported. We train two predictive models using a public cough dataset containing subjects with PCR- confirmed and patient-reported status. One model harnesses the data of PCR-confirmed subjects only, while the other - self-reported data only. We evaluate the performance of the two models and we observe a consistent performance improvement when the model is trained with PCR-confirmed data. We analyse the accuracy of the model, the stability of features exploited by the classifiers, and the performance of the model with limited training data.

Our results (i) highlight the need for using reliable data when training and evaluating COVID screening models, and (ii) indicate the need for more rigorous crowdsourcing practices for health data used to train machine learning classification models.

## Related work

Clinicians have been using sounds and acoustic data such as acoustic data to diagnose various conditions: voice pathologies, dry and wet cough, sleep disorders, and more^28–34^. Recently, several works also exploited sound data for large-scale COVID screening. In general, these utilised three types of sound data: cough, breathing, and speech.

Imran et al.^35^ implemented a COVID screening system that used the cough data recorded and transferred by a smartphone. Then, an AI model produced and returned the diagnostic prediction within two minutes. To attract researchers to work on COVID detection, Orlandic et al.^36^ created and shared a large COUGHVID dataset containing over 25,000 crowdsourced cough recordings. These covered a wide range of genders, ages, locations, and cough sounds. The work of Bader et al.^37^ proposed a model utilizing speech signal processing to screen COVID with cough data. Finally, Laguarta et al.^22^ proposed an AI model that allowed a large-scale pre-screening of COVID subjects.

Another stream of work aimed to identify COVID by analysing breathing data. The work of Wang et al.^38^ proposed a bidirectional neural network to achieve a large-scale screening of COVID subjects by analyzing breathing patterns. Similarly, Jiang et al.^39^ combined breathing data with thermal images to analyze the health status of individuals. However, this method required the subjects to wear a portable non-contact device including a thermal imaging camera. Instead of using breathing data on its own, some works predicted COVID cases by jointly analyzing cough and breathing data using various deep learning methods, such as recurrent neural networks (RNN)^40,41^.

Speech is another sound modality that can help detect COVID. For instance, Ismail et al. proposed a dynamic model analysing the movement of the vocal folds based on the observation that many symptomatic COVID patients have respiratory function impairments^42^. They hypothesised that such impairments affect vocal fold oscillation, and these changes can be harnessed to detect COVID. Ritwik et al. extracted Mel filter bank features from speech data to train an SVM classifier and classify COVID cases^43^. Likewise, Quartieri et al. exploited speech data to identify COVID symptomatic subjects using speech modelling techniques^44^.

While the previous studies used a range of data collection methods, sound data modalities, signal processing methodologies, and machine learning algorithms to train models and detect COVID subjects, the majority exploited data that was crowdsourced, meaning that the true infective status of patients is unclear. Second, the data collection settings (e.g., hardware and software in case of mobile apps) were likely to be diverse and not controlled. As has been shown for medical^45^ as well as broader machine learning models^46^, performance is substantially affected by the quality of the underlying data used to train them.

## Methodology

**Table 1:** Characterisation of the dataset.

Group (source)	Subjects/recordings	Duration (sec)	Coughs per subject
COVID (PCR-confirmed)	381	1.88	2.72
COVID (patient-reported)	114	1.84	2.37
Non-COVID (patient-reported)	203	3.07	3.79

We experimentally compare the performance of machine learning COVID cough classification models using either PCR-confirmed or patient-reported data. Hereafter, we refer to data provided by PCR-confirmed COVID subjects as *verified* data, while data provided by subjects with patient-reported infective status is referred to as *unverified*.

### Data

We use a publicly available dataset of COVID and non-COVID cough recordings^20^. The data contain 1322 cough recordings from as many subjects, collected by the MedInGroup primary health network. Two platforms were deployed for the data collection: call centre and Telegram mobile app. To eliminate potential differences associated with data collection and audio processing methods, we use only the 698 call centre recordings and disregard the Telegram recordings. Of these, MedInGroup verified positive infective COVID status of 381 subjects, which has been confirmed by a PCR test. The status of 114 COVID subjects was patient-reported, while all the 203 COVID negative subjects were patient-reported. Descriptive characterisation of the utilised data is provided in Table 1.

The performance of machine learning models is typically evaluated by partitioning the data into training and test sets. In our case, there are *two training sets*: PCR-confirmed COVID positive subjects and COVID negative subjects represent the training set with the verified data, and patient-reported and COVID negative subjects are another training set with the unverified data. However, we hold out a single *test set*, which contains only the PCR-confirmed COVID and non-COVID data.

The detailed steps of generating the training and test sets for evaluation purposes are as follows:

1. For the 203 non-COVID subjects, 160 are randomly selected for training and the remaining 43 – for testing.
2. For the 381 verified subjects, 114 are randomly selected for training and 20 randomly – for testing.
3. For the unverified data, all 114 patient-reported subjects are used for training.

In this way, the size of the verified and unverified training sets is identical. Each contains 274 subjects: 114 COVID subjects (PCR-confirmed or patient-reported) and 160 non-COVID subjects. The test set contains 63 subjects: 20 PCR-confirmed COVID subjects and 43 non-COVID subjects. To obtain solid empirical evidence, randomisation and partitioning of subjects was repeated five times and the results averaged.

### COVID Cough Classification

**Figure 1:**
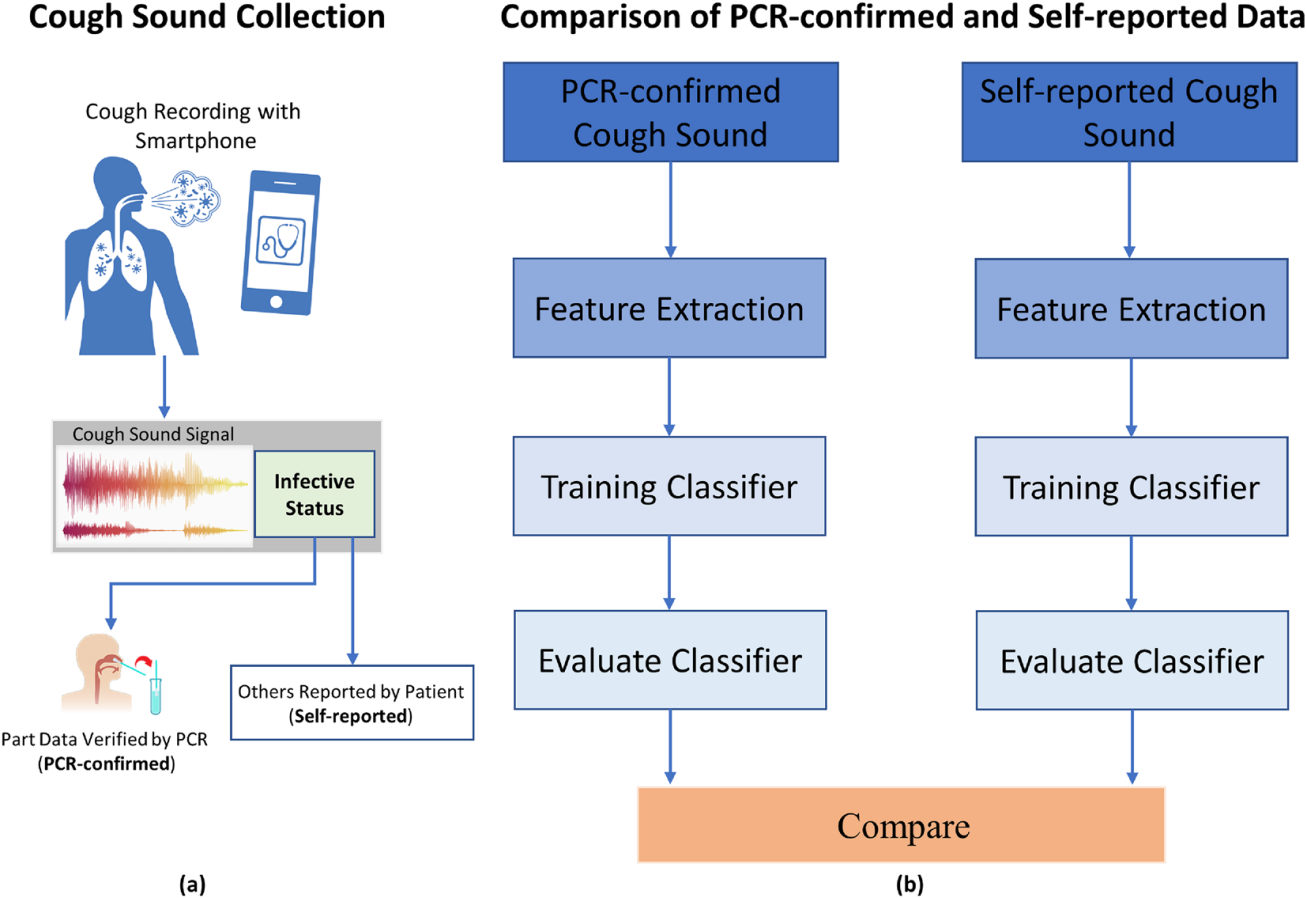
Methodology overview. (**a**) We utilize the cough sounds recorded by smartphones, where the infective status is either validated by a PCR test (PCR-confirmed) or reported by patients (self-reported). (**b**) We then compare the performance of identical classification frameworks harnessing these two types of sound. The frameworks include feature extraction, machine learning classifiers, and controlled evaluation setting.

A schematic overview of our method is shown in Fig. 1. We develop two classification frameworks, each using verified or unverified cough recording data. While the nature of the recordings is similar, the infective status label that accomppanies each recording is either PCR-confirmed or self-reported. The former is naturally more reliable than the latter. The subsequent classification framework is identical: each extracts informative features from the preprocessed recordings, performs feature selection to detect a subset of more predictive features, and classifies the recordings using a machine learning classifier, and evaluates the performance of the classifier. Since the two frameworks are identical, any performance differences observed should be attributed to differences in the input recording data.

#### Preprocessing and Feature Extraction

We first extract features from the recordings using an open-source openSMILE library^47^. openSMILE processes the input audio data in real-time. The recordings are converted into the WAV format for feature extraction with openSMILE. As a result, 6373 features are populated from each recording. To populate features, openSMILE extracts low-level descriptors (LLDs), which are combined with various filters and functionals. openSMILE offers various options for LLDs, including Waveform, FFT spectrum, and Mel/Bark spectrum. Upon the LLD extraction, the filters are utilised to smooth the feature contours. Since the length of the recordings varies, polynomial regression, and transformations are applied to standardize the feature length.

#### Feature Selection

Due to the high dimensionality of features produced by openSMILE, some features may contain redundant and noisy data, which is likely to degrade the accuracy of the classifiers. To minimize the risks of overfitting, we use ElasticNet^48^ for feature selection to identify data features that are predictive of infectious status, whilst noisy and redundant ones are discarded. The parameterization of ElasticNet is based on offline experiments that are not reported.

#### Classification Models

Once the predictive features are selected, we feed these into classifiers to predict the subjects’ COVID status. To ensure our results are generalizable to a broad variety of machine learning approaches, we tested a portfolio of seven binary classifiers that belong to three families: two *statistical classifiers* (Logistic Regression (LR)^49^ and Linear Discriminative Analysis (LDA)^50,51^), two *ensemble-based classifiers* (Random Forest (RF)^52^ and Gradient Boosting Classifier (XGB)^53^), and three *deep learning classifiers* (Deep Neural Network (DNN)^54^, CNN-RNN^55^ and Contextual Attention CNN (CA-CNN)^56^). LR and LDA exploit statistical inference to produce the probability of an instance being a member of each class. RF and XGB represent ensembles of predictive models, where each model classifies independently and the predictions of the models are integrated. DNN is a deep network with three hidden layers between the input and output layers. The sizes of the hidden layers are 3000, 1000 and 200, respectively. To avoid over-fitting, and for efficient gradient propagation, we include dropout and ReLU after each layer. CNN-RNN and CA-CNN are modern deep learning methods, exploiting hybrid networks and attention mechanism for cough data classification. We run CNN-RNN and CA-CNN on one Telsla T4 GPU, while the other classifiers were trained and evaluated on CPUs.

#### Metrics

The performance of classification models was evaluated using the accuracy (Acc) metric, e.g. the fraction of COVID and non-COVID subjects that are correctly classified. As shown in Table 1, the datasets are often imbalanced with respect to the number of COVID and non-COVID subjects. To obtain reliable performance metrics, we also compute the area under the receiver operating characteristic curve (AUC). These metrics are averaged across five runs for five data partitions.

## Results

### COVID vs non-COVID classification

The classification models were trained independently on the two input datasets and their ability to predict COVID infective status using the test set is reported in Table 2. The accuracy (Acc) and AUC of each classifier is given for each of the five runs alongside with the averaged performance. The highest accuracy and AUC scores are highlighted in bold.

It can be seen that for all classifiers, the average accuracy and AUC scores of the models trained using the verified data are superior or equal to those of the models using unverified data. The differences between the classifiers trained using the two source of data – average accuracy difference up to 0.06 and AUC difference in the 0.02-0.10 range – are consistent across the classifiers. Overall, the highest AUC=0.83 is achieved by RF and the highest Acc=0.78 is achieved by CA-CNN.

As the number of COVID and non-COVID subjects was imbalanced, the AUC metric more reliably represents classification performance than the average accuracy. Considering the AUC scores, we observe the deep learning methods, CA-CNN and CNN-RNN, do not perform better than statistical methods. This can potentially be explained by the scarce training data, not allowing to train accurate deep networks. Likewise, a simple method like LR expectedly does not exhibit a strong performance. The ensemble methods, RF and XGB, outperform other methods, due to their reliance on multiple classifiers, which makes the prediction more stable and robust.

**Table 2:** Accuracy and AUC for COVID vs non-COVID classification.

Run	Training data	LR		RF		XGB		DNN		LDA		CA-CNN		CNN-RNN	
		Acc	AUC	Acc	AUC	Acc	AUC	Acc	AUC	Acc	AUC	Acc	AUC	Acc	AUC
1	Verified	0.77	0.81	0.82	0.85	0.80	0.80	0.65	0.70	0.75	0.82	0.75	0.77	0.77	0.76
	Unverified	0.67	0.60	0.75	0.79	0.73	0.72	0.45	0.68	0.75	0.75	0.57	0.58	0.60	0.51
2	Verified	0.65	0.68	0.58	0.69	0.67	0.68	0.72	0.74	0.68	0.72	0.8	0.81	0.73	0.80
	Unverified	0.63	0.65	0.70	0.73	0.73	0.70	0.68	0.75	0.77	0.84	0.43	0.34	0.63	0.58
3	Verified	0.75	0.83	0.87	0.94	0.80	0.90	0.67	0.69	0.80	0.91	0.78	0.82	0.75	0.86
	Unverified	0.70	0.74	0.83	0.91	0.70	0.77	0.48	0.55	0.80	0.86	0.67	0.60	0.37	0.59
4	Verified	0.58	0.52	0.83	0.90	0.77	0.79	0.67	0.73	0.77	0.84	0.78	0.78	0.75	0.87
	Unverified	0.62	0.56	0.73	0.81	0.68	0.56	0.68	0.75	0.70	0.76	0.40	0.33	0.32	0.32
5	Verified	0.62	0.67	0.62	0.76	0.73	0.80	0.63	0.66	0.63	0.72	0.78	0.82	0.68	0.69
	Unverified	0.65	0.73	0.67	0.71	0.70	0.68	0.73	0.66	0.63	0.70	0.43	0.51	0.60	0.54
Average	Verified	**0.67**	**0.70**	**0.74**	**0.83**	**0.75**	**0.79**	**0.67**	**0.70**	**0.73**	**0.80**	**0.78**	**0.80**	**0.74**	**0.80**
	Unverified	0.65	0.66	**0.74**	0.79	0.71	0.69	0.61	0.68	**0.73**	0.78	0.50	0.47	0.50	0.51

Best results are indicated in boldface.

**Figure 2:**
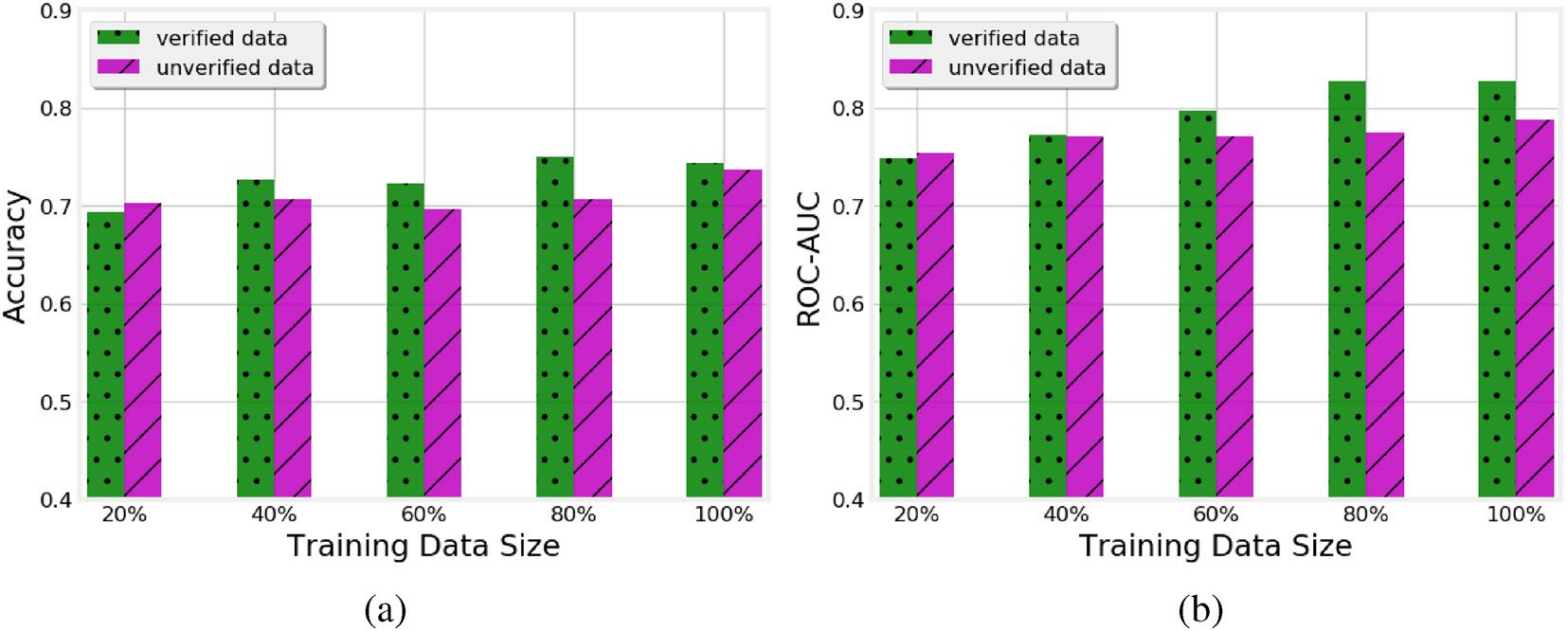
Impact of the volume of verified and unverified training data on (**a**) accuracy and (**b**) AUC of the RF classifier

### Effects of Training Data Size

In this experiment, we study the impact of the volume of the available training data on the accuracy of the verified and unverified classifiers using RF, which was the best performing of the classifiers. We vary the percentage of training data in each run to 20%, 40%, 60%, and 80% of the original training data. The accuracy and AUC scores of each increment in size are shown in Fig. 2.

As one would expect, the performance of the classifier generally improves as more training data becomes available. Focusing on the comparison between verified and unverified models, we note that their performance is comparable with 20% and 40% of the training data. However, both the Acc and AUC of the verified model are better than those of the unverified model when 60%, 80%, and 100% of data is exploited. This indicates that the unverified models do not benefit from the additional training data as much as the verified ones and likely need more data to improve their performance.

**Figure 3:**
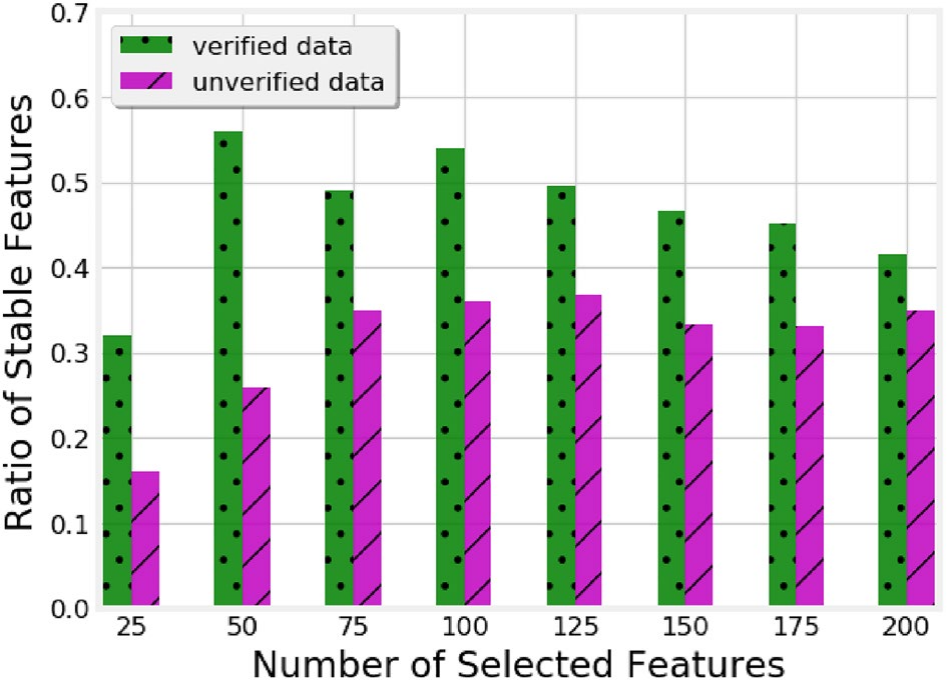
Ratio of stable features among the features selected by models trained on verified and unverified data.

**Figure 4:**
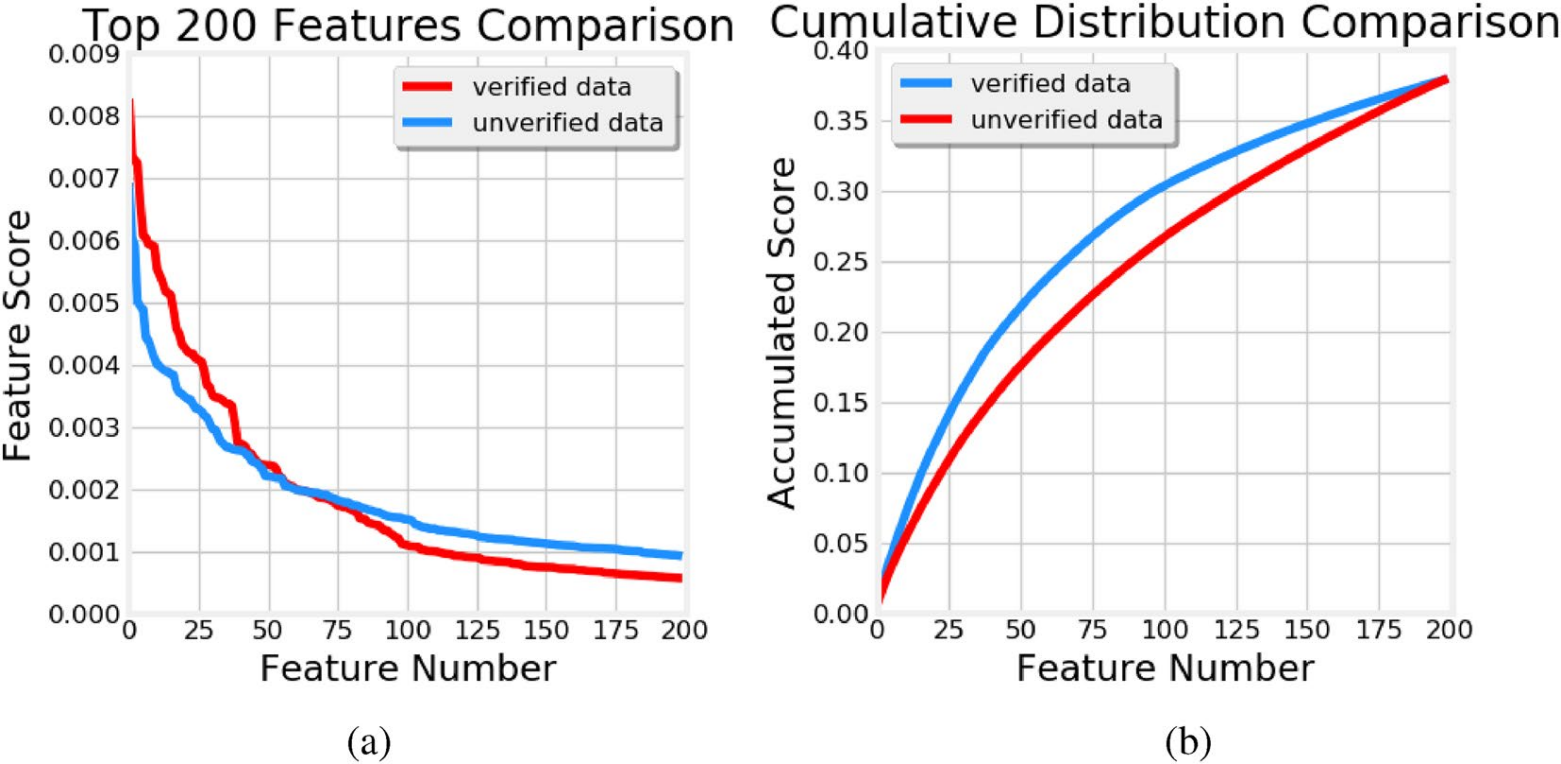
(**a**) Weights and (**b**) cumulative distribution of the weights of top-200 features

### Stability of Features

Next, we analyze the top-200 features produced by the RF classifier, in the verified and unverified models. For this, we define the notion of *stable* features as the subset of features present in top-*k* features across all the five runs and compute the ratio of stable features by dividing the number of stable features by *k*. We increase *k* from 25 to 200, compute for each *k* the ratio of stable features, and plot this in Fig. 3. As can be seen, the ratio of stable features for the verified models is consistently higher than for the unverified ones and the difference between the two is more prominent for low values of *k*. This shows that the verified data allows training more stable models, potentially offering a better generalisability.

This trend is further supported in Fig. 4 where we plot the weights (**a**) and cumulative distribution of the weights (**b**) of top-200 features for the verified and unverified models. As expected, the weights exhibit a long-tail distribution, with a few features dominating the predictions. Out of the 6373 features extracted by openSMILE in the verified model, top-25 features account for as much as 15% of the overall weight and top-200 features account for 38%. Additionally, it is evident that the 60 heaviest features of the verified model have consistently higher weights than the corresponding 60 features of the unverified model. Hence, the former are likely to be seen as more informative and predictive than the latter.

### Distinguishing PCR-confirmed vs patient-reported COVID classification

Finally, we set out to see if any of the hypothesized differences between PCR-confirmed and self-reported data could be detected. To this end, we trained an RF classifier using the 114 PCR-confirmed subjects and 114 self-reported positive subjects. We measured the ability of the RF classifier to detect whether a subject was from the PCR-confirmed or self-reported group in a 5-fold cross validation experiment.

**Table 3:** Verified versus unverified COVID subject classification.

Run	RF-Acc	RF-AUC
1	0.75	0.82
2	0.68	0.72
3	0.80	0.91
4	0.77	0.84
5	0.63	0.72
Average	0.73	0.80

As reported in Table 3, the accuracy and AUC of RF for each fold are relatively high, reaching AUC as high as 0.91. Overall, RF achieves mean Acc=0.73 and mean AUC=0.8 across the five folds. These results indicate that RF can accurately distinguish between the two inputs, even though contributed by seemingly COVID positive subjects. Hence, we posit that the cough recording of PCR-confirmed COVID subjects may differ from those of subjects with patient-reported infective status, as the classifier successfully differentiates between them.

## Discussion

In this work, we set out to assess the value of using reliable training data for machine learning based COVID-19 screening using cough data. We hypothesized that the less certain infective status of subjects providing patient-reported COVID status with no PCR test could degrade the performance of the predictive models and its ability to accurately screen COVID subjects. Hence, we compared the accuracy and AUC achieved by classification models trained using cough sounds provided by subjects with PCR-confirmed infective COVID status to the models built using self-reported data.

We report several experiments, which compare the performance of identical classification models trained on either data provided by PCR-confirmed or self-reported subjects data, from several perspectives. These demonstrate that models trained with the more reliable PCR-confirmed data achieve a higher accuracy than those trained with patient-reported data. This finding holds for all the classifiers we experimented with. Moreover, classifier trained on the PCR-confirmed data is found to require less data than the one trained on patient-reported data. The performance of the former stabilises at 60%-80% of the available training data, while the latter remains data-hungry and not as accurate. Notably, we observe that the classifier using PCR-confirmed data rely on a smaller set of more stable predictive features.

Analyzing the nuanced selection of training data harnessed by the classifiers (see the Data sub-section of the Methodology section), comparable characteristics of the training datasets (see Table 1), and rigorous evaluation methodology (see the stable results across the runs in Table 2), we question the inherent reliability of the two input datasets, as the only evident point of differentiation between the classifiers. Considering all the above, we posit that the better performance of models harnessing the verified data of PCR-confirmed subjects should be attributed to the more reliable nature of such data. Hence, we believe that the observed differences are caused by a smaller number of mis-labelled subjects in the verified data. Used as the more reliable training data labels, they allow the classifier to more accurately learn the features characterising COVID cough sounds and achieve more accurate predictions for the test data.

While this noise manifests in our experiments only in COVID cough sounds and infective status, our findings raise important questions around the *use of patient-reported data for training clinical decision-support*. It is evident that collecting abundant and reliable data often requires expensive clinician examination or confirmatory molecular tests as gold standard. Collecting such a data at scale and feeding it into machine learning models may raise patient data privacy concerns and entail data linkage considerations^57^. Moreover, the data may just not be readily available for rare conditions. Relying on data voluntarily contributed by patients may allay these concerns and offer an appealing alternative. However, as our experiments demonstrate, such data is prone to noises, which may yield sub-standard performance and unreliable decision-support.

To illustrate the differences between the data provided by PCR-confirmed and self-reported subjects, in the last experiment we trained the RF classifier to distinguish between the two. Notably, the classifier successfully learned the differences, achieving mean AUC of 0.8. This indicates that the data of the two cohorts of subjects is far from being identical. Hence, we emphasize that although patient-reported COVID status is naturally easier to obtain, special caution is required when using this information as a proxy for the actual infective status. Acknowledging that the majority of prior works on machine learning based methods for COVID cough classification utilized crowdsourced data and patient-reported infective status to train the developed algorithms, we note that the validity of results obtained in these works remains unclear.

Our work is not without limitations. First, the infective status of non-COVID subjects was patient-reported and not verified by PCR. It is possible that some of them were either asymptomatic or had recovered from COVID before the data collection. Despite not having symptoms at the data collection time, their respiratory system might have been affected by COVID, which could bias the classifier. In order to obtain more sound evidence, we would like to partition the non-COVID class into PCR-confirmed and patient-reported subjects, similarly to the COVID class and then revisit the validity of our findings.

Second, the experiments involved a relatively small cohort of approximately 700 subjects. While collecting audio recordings is fairly straightforward with the ubiquity of smartphones, obtaining reliable PCR-confirmed status of COVID subjects is encumbered by privacy and confidentiality issues. Hence, it is unclear whether our findings will generalise for a larger and more diverse population. To address this, we propose to integrate audio data collection into the PCR testing procedures, following explicit consent of the patients and deployment of appropriate privacy-preserving technologies. This would facilitate future larger-scale replication studies.

Third, the utilized dataset included no medical information beyond COVID status. In particular, no information about the stage of COVID, period of time since the positive PCR test, or severity of the disease was available. Each of these is a factor that can potentially affect the respiratory system, cough recordings, and, in turn, the performance of the classifier. Likewise, no information about co-morbidities of the subjects, which could have affected their health status was available. Hence, we could not control for any of these factors, although they potentially further biased the classifier. We posit that collecting and harnessing this information will diminish the dependence of the classifier on the cough recordings and potentially unreliable infective status.

## Conclusion

In this work, we investigated the reliability of patient-reported data utilized for the purposes of screening COVID-19 subjects. While our results supported previous works that demonstrated high predictive accuracy, we observed that the reliability of data used to train the machine learning based models plays a crucial role, with the models trained on patient-reported data demonstrating inferior performance to those trained with the more reliable PCR-confirmed data.

Whilst verified clinical data are harder to obtain, often require clinician involvement or pathology tests, and may entail privacy and confidentiality considerations, it practically improves the performance of the machine learning models. This emphasises that reliable outcome measures are imperative for the accuracy of COVID detection technologies and clinical decision-support more generally.

## Data Availability

All experimental data can be found at https://github.com/covid19-cough/dataset.
